# COVID-19 case fatality risk by age and gender in a high testing setting in Latin America: Chile, March–August 2020

**DOI:** 10.1186/s40249-020-00785-1

**Published:** 2021-02-03

**Authors:** Eduardo A. Undurraga, Gerardo Chowell, Kenji Mizumoto

**Affiliations:** 1 Escuela de Gobierno, Pontificia Universidad Católica de Chile, Vicuña Mackenna 4860, Macul, CP 7820436 Santiago, Región Metropolitana Chile; 2 Millennium Initiative for Collaborative Research in Bacterial Resistance (MICROB-R), Santiago, Chile; 3 Department of Population Health Sciences, School of Public Health, Georgia State University, Atlanta, GA USA; 4 Graduate School of Advanced Integrated Studies in Human Survivability, Kyoto University Yoshida-Nakaadachi-Cho, Sakyo-ku, Kyoto, Japan; 5 Hakubi Center for Advanced Research, Kyoto University, Yoshidahonmachi, Sakyo-ku, Kyoto, Japan

**Keywords:** COVID-19, Chile, Death risk by age group, Time-delay adjusted case fatality rate, Latin America

## Abstract

**Background:**

Early severity estimates of coronavirus disease 2019 (COVID-19) are critically needed to assess the potential impact of the ongoing pandemic in different demographic groups. Here we estimate the real-time delay-adjusted case fatality rate across nine age groups by gender in Chile, the country with the highest testing rate for COVID-19 in Latin America.

**Methods:**

We used a publicly available real-time daily series of age-stratified COVID-19 cases and deaths reported by the Ministry of Health in Chile from the beginning of the epidemic in March through August 31, 2020. We used a robust likelihood function and a delay distribution to estimate real-time delay-adjusted case-fatality risk and estimate model parameters using a Monte Carlo Markov Chain in a Bayesian framework.

**Results:**

As of August 31, 2020, our estimates of the time-delay adjusted case fatality rate (CFR) for men and women are 4.16% [95% Credible Interval (CrI): 4.09–4.24%] and 3.26% (95% CrI: 3.19–3.34%), respectively, while the overall estimate is 3.72% (95% CrI: 3.67–3.78%). Seniors aged 80 years and over have an adjusted CFR of 56.82% (95% CrI: 55.25–58.34%) for men and 41.10% (95% CrI: 40.02–42.26%) for women. Results showed a peak in estimated CFR during the June peak of the epidemic. The peak possibly reflects insufficient laboratory capacity, as illustrated by high test positivity rates (33% positive 7-day average nationally in June), which may have resulted in lower reporting rates.

**Conclusions:**

Severity estimates from COVID-19 in Chile suggest that male seniors, especially among those aged ≥ 70 years, are being disproportionately affected by the pandemic, a finding consistent with other regions. The ongoing pandemic is imposing a high death toll in South America, and Chile has one of the highest reported mortality rates globally thus far. These real-time estimates may help inform public health officials' decisions in the region and underscore the need to implement more effective measures to ameliorate fatality.

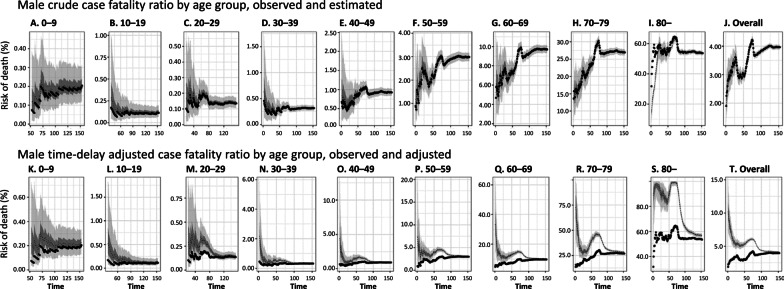

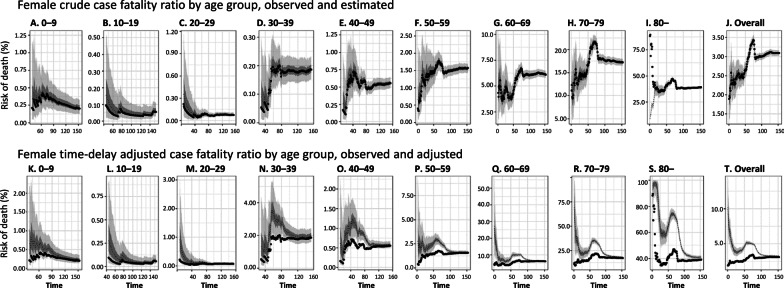

## Background

The coronavirus disease 2019 (COVID-19) pandemic has strained or overwhelmed health systems across the world [1, 2], with about 25.5 million COVID-19 cases and 850 thousand deaths as of August 31, 2020 [3, 4]. The first case in South America of severe acute respiratory syndrome coronavirus 2 (SARS-CoV-2) infection, the cause of COVID-19, was reported on February 25 in São Paulo, Brazil, a travel hub in the region [5]. A few weeks later, countries in the region had imposed major epidemic control measures, such as closed borders, restricted travel, closure of schools and universities, and enforced lockdowns [6, 7]. Despite these measures, the ongoing coronavirus pandemic has already imposed a high toll on most countries in South America, killing thousands in Brazil (121 100), Peru (28 800), Colombia (19 700), Chile (15 700), Argentina (8700), Ecuador (6600), and Bolivia (5000), thus far [3]. In addition to already-strained healthcare systems, other factors have affected the dynamics of the pandemic in the region, including migration, sociopolitical crises, struggling economies, other infectious disease outbreaks, poor leadership, and challenges tied to the implementation of social distancing, hygiene, and lockdown strategies, due to inadequate water and sanitation infrastructure and precarious living conditions [6, 8–15].

The Ministry of Health reported the first COVID-19 case in Chile on March 3, 2020 [16]. The government activated a quick response effort, announcing restrictions on large gatherings on March 13, and subsequently, closure of all daycares, schools, and universities (March 16), border controls, telework recommendations (March 18), closure of non-essential businesses (March 19), national night curfews (March 22), and a strategy of intermittent localized lockdowns in selected municipalities since March 26 [17]. Estimates for the early stage of the epidemic showed sustained transmission with an estimated reproduction number *R*_0_ = 1.8 [95% confidence interval (*CI*): 1.6–1.9] [18]. Research suggests that containment measures at the start of the epidemic significantly slowed down the spread of the virus in Chile [18]. Early models estimated that a surge in the number of ill patients could overwhelm treatment capacity by June unless aggressive control measures were implemented [19]. Control measures prevented the epidemic's growth in some regions, particularly during the first months of the epidemic [20–22].

Chile saw a rapid increase in reported COVID-19 cases during May, reaching a peak 7-day average incidence of about 6000 cases per day (31 per 100 000 population) by mid-June [16]. Health system capacity reached saturation levels by the end of June, with 95% intensive care unit occupancy in Greater Santiago and 89% at the national level [23, 24]. As of August 31, the Ministry of Health’s surveillance system EPIVIGILA has recorded a total of 444 921 COVID-19 cases and 15 756 deaths [16, 25]. Most of these reported COVID-19 cases and deaths have been confirmed by reverse transcription-polymerase chain reaction (RT-PCR) assay. However, these reported cases and deaths also include some clinically diagnosed COVID-19, particularly during the peak of the epidemic when the healthcare system reached saturation [23], and laboratories could not satisfy the demand for diagnostic tests. In June, 7-day average positivity rates of the RT-PCR tests reached 33% nationally. Evidence shows that the circulation of other respiratory viruses, such as influenza, has been negligible in Chile and elsewhere in the southern hemisphere [26]. With 130 total RT-PCR tests per 1000 population by August 31, Chile has tested at a higher rate than any other Latin American country [27, 28].

During the first two months of the epidemic, the crude case fatality rate (CFR), the number of cumulative deaths over the number of cumulative cases, in Chile (1.2%) remained well below the global average (6.5%) [4, 16]. Because initial COVID-19 cases in Chile occurred among relatively low-risk groups (20–60 years of age), several infectious disease experts warned about authorities’ excessive confidence over early success [29]. The CFR is a commonly used estimate of the severity of an epidemic [30]. It provides a reliable benchmark for public health officials to decide the intensity and duration of interventions to mitigate or suppress an epidemic [31]. Nevertheless, obtaining CFR estimates during an epidemic is challenging, as CFR is typically affected by right censoring and ascertainment bias [32–36]. Right-censoring occurs because of a time delay between the onset of symptoms and death, thus underestimating CFR. Under-ascertainment of cases occurs because mild or asymptomatic COVID-19 cases often go undetected by disease surveillance systems, which are not designed to detect all infections [33, 37, 38]. SARS-CoV-2 infection can result in a broad spectrum of clinical outcomes, including asymptomatic infection, mild symptoms, hospitalization, or death [39–41].

Here we provide real-time estimates of adjusted age-specific CFR during the COVID-19 epidemic in Chile, March through August 2020, to gauge the severity of the SARS-CoV-2 epidemic. These CFR estimates could inform critical decisions by public health officials in Chile and Latin America.

## Methods

### Data sources

We obtained daily cumulative numbers of reported COVID-19 cases and deaths stratified by age group and gender from March 3, 2020, through August 31, 2020, from the Ministry of Health’s surveillance system EPIVIGILA [16, 25]. The datasets used are available from the Ministry of Science’s COVID-19 repository [25]. COVID-19 death definition follows the World Health Organization’s updated guidelines for COVID-19 based on the International Statistical Classification of Diseases and Related Health Problems (ICD) standards [42]. Different groups had different starting times in the time series, which correspond to the day when the first death was reported.

### Statistical analysis

For the estimation of CFR in real-time, we employed the delay from hospitalization to death, *h*_*s*_. We assumed *h*_*s*_ = *H(s) – H(s-1)* for *s* > *0* where *H(s)* is a cumulative density function of the delay from hospitalization to death and follows a gamma distribution with mean 10.1 days and standard deviation (SD) 5.4 days [43]. Let π_*a,ti*_ be the time-delay adjusted case fatality ratio on reported day t_*i*_ in area a, the likelihood function of the estimate π_*a,ti*_ is

$$\left( {\pi_{{a,t_{i} }} ;c_{a,t} ,D_{a,t} } \right) = \mathop \prod \limits_{{t_{i} }} \left( {\begin{array}{*{20}c} {\mathop \sum \limits_{t = 1}^{{t_{i} }} c_{a,t} } \\ {D_{{a,t_{i} }} } \\ \end{array} } \right)\left( {\pi_{{a,t_{i} }} \frac{{\mathop \sum \nolimits_{t = 2}^{{t_{i} }} \mathop \sum \nolimits_{s = 1}^{t - 1} c_{a,t - s} h_{s} }}{{\mathop \sum \nolimits_{t = 1}^{{t_{i} }} c_{a,t} }}} \right)^{{D_{{a,t_{i} }} }} \left( {1 - \pi_{{a,t_{i} }} \frac{{\mathop \sum \nolimits_{t = 2}^{{t_{i} }} \mathop \sum \nolimits_{s = 1}^{t - 1} c_{a,t - s} h_{s} }}{{\mathop \sum \nolimits_{t = 1}^{{t_{i} }} c_{a,t} }}} \right)^{{\mathop \sum \limits_{t = 1}^{ti} c_{a,t} - D_{{a,t_{i} }} }}$$

where *c*_*a,t*_ represents the number of new cases with reported day *t* in area *a*, and *D*_*a,ti*_ is the cumulative number of deaths until reported day t_*i*_ in an area *a* [44, 45]. Among the cumulative cases with reported day *t* in area *a, D*_*a,ti*_ have died, and the remainder have survived the infection. The contribution of those who have died with biased death risk is shown in the middle parenthetical term. The contribution of survivors is presented in the right parenthetical term. We assume that *D*_*a,ti*_ is the result of the binomial sampling process with probability *π*_*a,ti*_.

We estimated model parameters using a Monte Carlo Markov Chain (MCMC) method in a Bayesian framework. We evaluated the convergence of MCMC chains using the potential scale reduction statistic [46, 47]. Estimates and 95% credibility intervals (CrI) for these estimates are based on each parameter's posterior probability distribution and based on the samples drawn from the posterior distributions.

All statistical analyses were conducted in R version 3.6.1 (R Foundation for Statistical Computing, Vienna, Austria) using the ‘rstan’ package. This research was considered exempt from ethical review because it is limited to secondary data analysis based on publicly available data [16, 25], which contains no information that can directly or indirectly identify an individual. All data used in this analysis are publicly available [25].

## Results

### Epidemiological characterization of COVID-19 in Chile

**Fig. 1:**
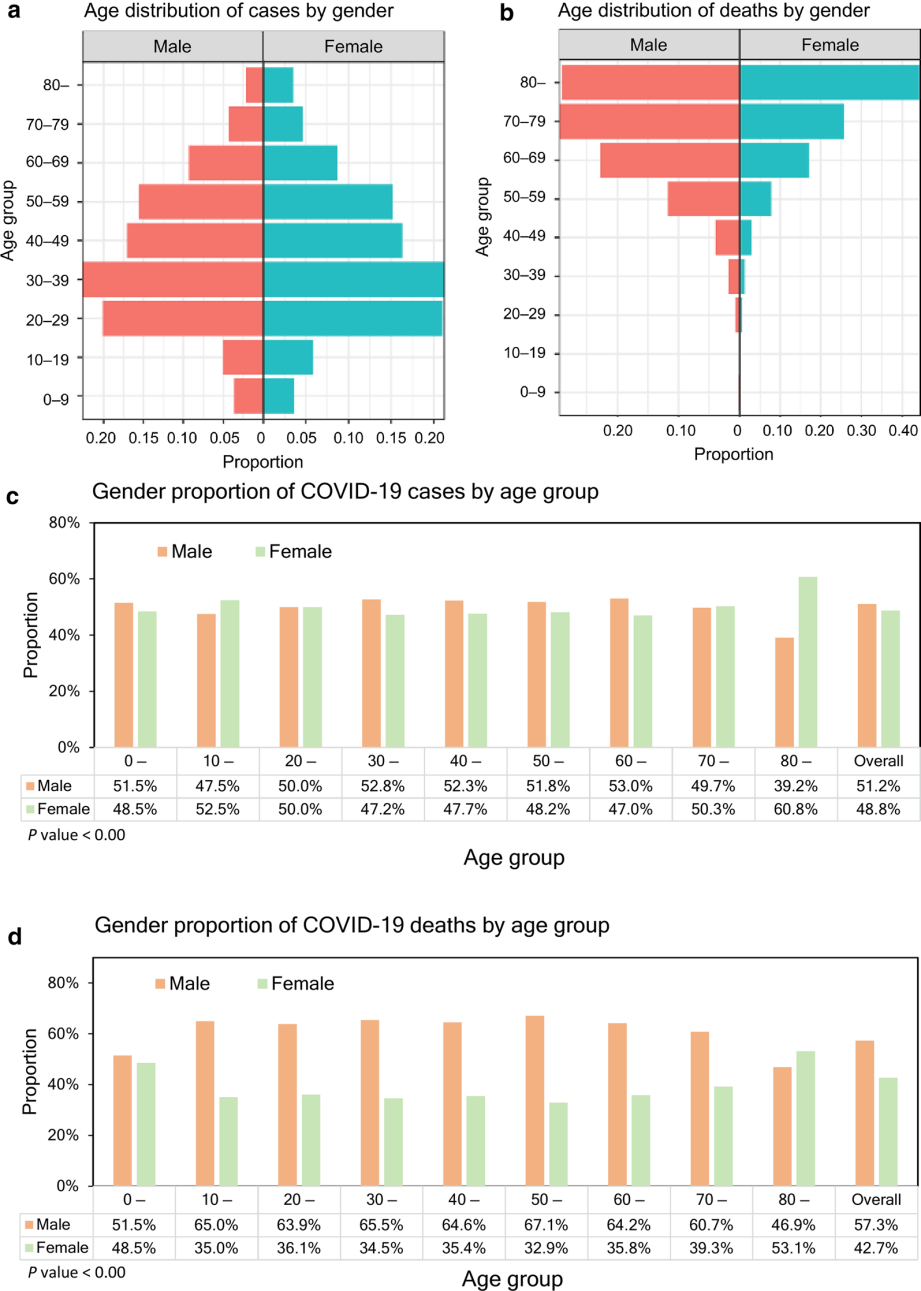

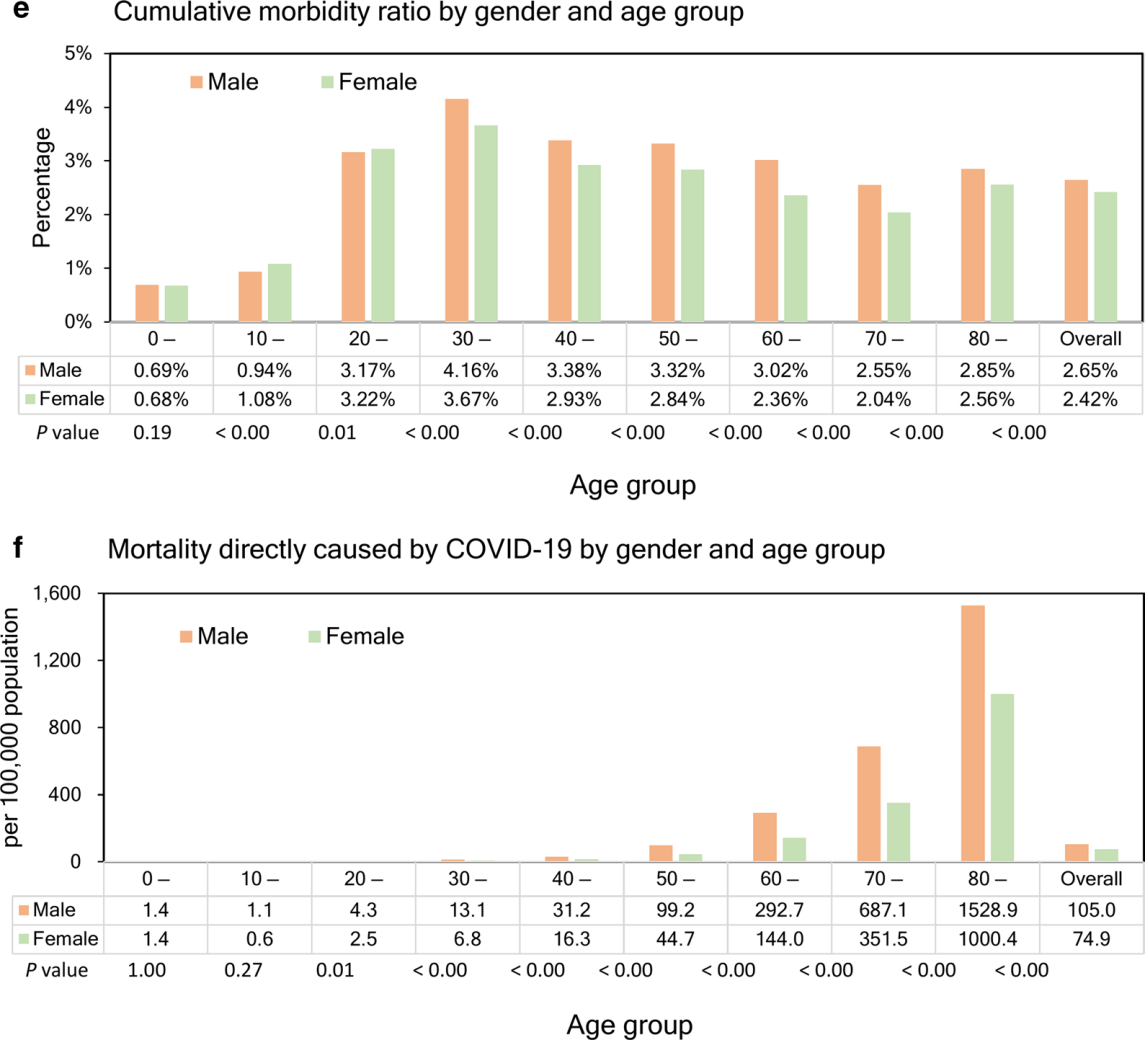
Epidemiological characterization of COVID-19 in Chile, as of August 31, 2020. **a** Age distribution of reported cases by gender. **b** Age distribution of reported deaths by gender. **c** Gender proportion of COVID-19 cases by age group. **d** Gender proportion of COVID-19 deaths by age group. **e** Cumulative morbidity risk by gender and age group. **f** Mortality directly caused by COVID-19 by gender and age group

The Ministry of Health has reported 444 921 COVID-19 cases and 15 756 deaths as of August 31 [16, 25]. For men, most reported cases were persons aged 30–39 years (22.7%), followed by 20–29 year-olds (20.1%) and 40–49 year-olds (17.1%) (Table 1). Most reported deaths were seniors, especially 70–79 year-olds (29.5%), followed by those aged 80 years and older (29.2%), and 60–69 year-olds (22.8%) (Table 1, Fig. 1a, b). We found a similar pattern for women, except that most deaths were reported among women aged 80 years and older (44.4%).

**Table 1:** Distribution of the COVID-19 cases in Chile by sex and age groups, as of August 31, 2020

Age group	Men				Women			
	Cases	Deaths	cCFR (%)	Mortality per 100 000 population	Cases	Deaths	cCFR (%)	Mortality per 100 000 population
	(%)	(%)			(%)	(%)		
All	227 642	9035	3.97	105.0	217 279	6721	3.09	74.9
	(100)	(100)			(100)	(100)		
0–9	8357	17	0.20	1.4	7864	16	0.20	1.4
	(3.7)	(0.2)			(3.6)	(0.2)		
10–19	11 505	13	0.11	1.1	12 692	7	0.06	0.6
	(5.1)	(0.1)			(5.8)	(0.1)		
20–29	45 736	62	0.14	4.3	45 649	35	0.08	2.5
	(20.1)	(0.7)			(21.0)	(0.5)		
30–39	51 596	163	0.32	13.1	46 201	86	0.19	6.8
	(22.7)	(1.8)			(21.3)	(1.3)		
40–49	38 870	359	0.92	31.2	35 426	197	0.56	16.3
	(17.1)	(4.0)			(16.3)	(2.9)		
50–59	35 543	1061	2.99	99.2	33 012	520	1.58	44.7
	(15.6)	(11.7)			(15.2)	(7.7)		
60–69	21 215	2057	9.70	292.7	18 788	1148	6.11	144.0
	(9.3)	(22.8)			(8.6)	(17.1)		
70–79	9908	2669	26.94	687.1	10 015	1726	17.23	351.5
	(4.4)	(29.5)			(4.6)	(25.7)		
80–	4912	2634	53.62	1528.9	7632	2986	39.12	1000.4
	(2.2)	(29.2)			(3.5)	(44.4)		

*cCFR* Crude case fatality rate

The proportion of male cases is higher than 50% across age groups, except for 10–19 year-olds, 70–79 year-olds, and those aged 80 years and above (*χ*^2^ test, *P*-value < 0.0001, Fig. 1c). The proportion of male deaths is higher than 50% except for those aged 80 years and above (*χ*^2^ test, *P* < 0.0001, Fig. 1d). Figure 1e presents the cumulative morbidity ratio by gender and age group. The ratio of illness among males is significantly higher than among females across most age groups (proportion test, *P* < 0.000), except for 10–19 year-olds (proportion test, *P* < 0.00) and 20–29 year-olds (proportion test, *P* = 0.01). We found no statistically significant differences for 0–9 year-olds (proportion test, *P* = 0.19).

We found that mortality per 100 000 population directly caused by COVID-19 among males is higher than among females for all age groups 20 years and above (proportion test, *P* < 0.05, Fig. 1f). We found no statistically significant differences for individuals aged 0–19 years.

### Evolution of cases and deaths by age group

**Fig. 2:**
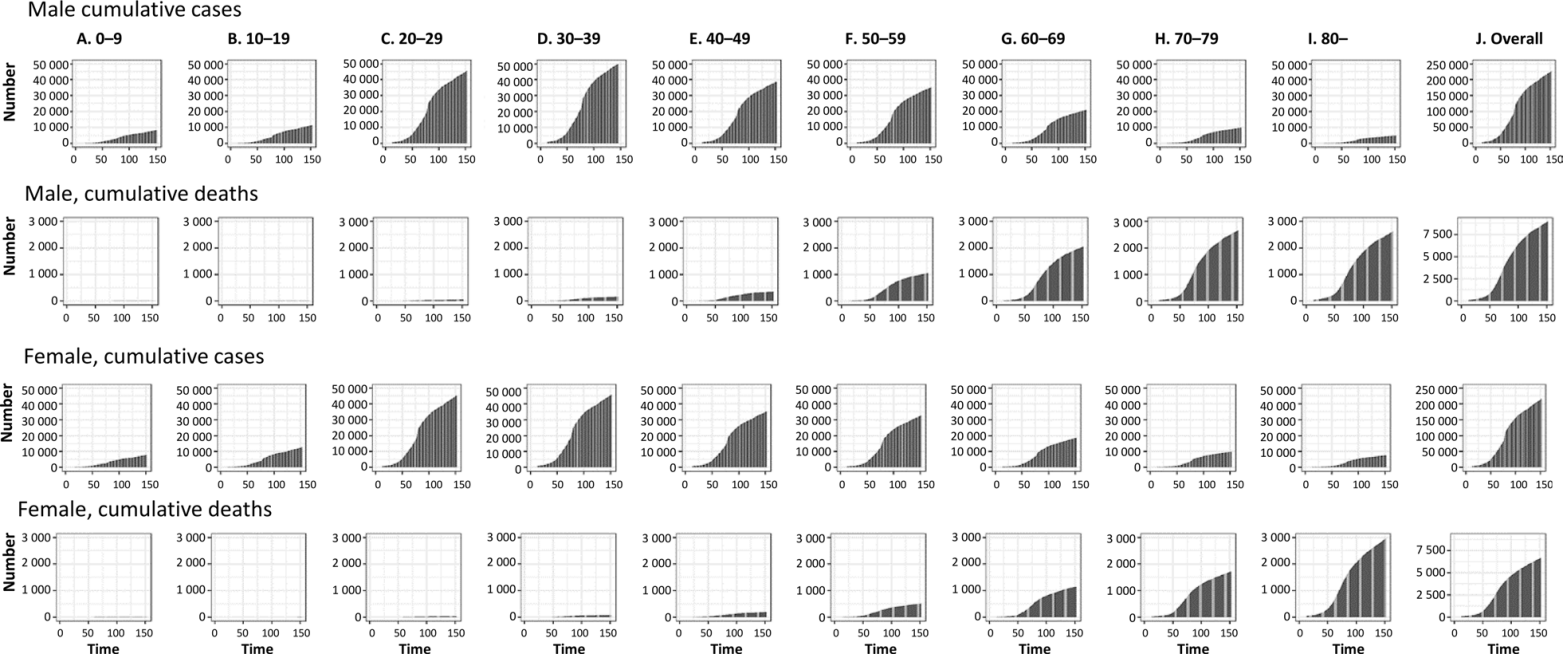
Temporal distribution of COVID-19 cases and deaths by age group, April through August 31, 2020, Chile. Cumulative cases and deaths of COVID-19 for men (first and second rows) and women (third and fourth rows) for the following age groups **a** 0–9, **b** 10–19, **c** 20–29, **d** 30–39, **e** 40–49, **f** 50–59, **g** 60–69, **h** 70–79, **i** 80 years or more, (N) all fatal cases over time. Day 1 corresponds to April 1 in 2020. Because the dates of illness onset were not available, we used dates of reporting by the Ministry of Health

The reported cumulative COVID-19 cases and deaths by age group and gender (A through J) over time are shown in Fig. 2. The figure suggests cumulative cases of COVID-19 are growing faster than cumulative deaths. The growth curve for cumulative cases across all age groups increases exponentially after around day 30 (April 30, 2020) until around day 80 (June 19, 2020). Exponential growth in cumulative deaths for all age groups occurs approximately after day 45 (May 15, 2020).

### Crude and time-delay adjusted risk of death

**Fig. 3:**
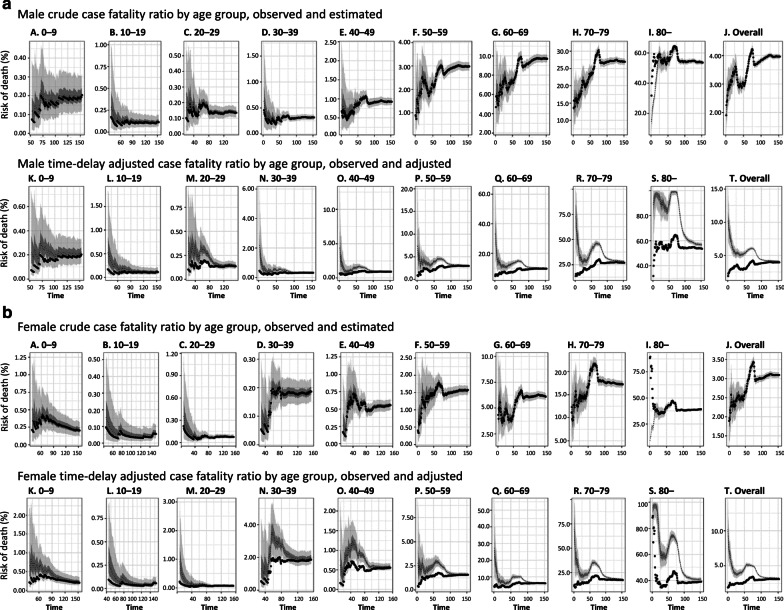
Temporal variation of risk of death caused by COVID-19, April through August 19, 2020, Chile. Upper two rows; Male risk of death, Lower two rows; Female risk of death. Observed and posterior estimated of crude case fatality ratio of persons aged **a** 0–9, **b** 10–19, **c** 20–29, **d** 30–39, **e** 40–49, **f** 50–59, **g** 60–69, **h** 70–79, **i** 80 and more, and **j** all age groups. Time-delay adjusted case fatality ratio of persons aged **k** 0–9, **o** 40–49, **p** 50–59, **q** 60–69, **r** 70–79, **s** 80 and more, and **t** all age groups. Day 1 corresponds to April 1, 2020. Black dots show crude case fatality ratio, and light and dark indicate 95% and 50% credible intervals for posterior estimates, respectively

The observed and model-based posterior estimates of the crude CFR of COVID-19 by age group (A–J) and time-delay adjusted CFR by age group (K–T) are shown in Fig. 3. Black dots show crude case-fatality ratios, and light and dark indicate 95% and 50% credible intervals for posterior estimates, respectively.

Overall, our model-based crude CFR fitted the observed data well. Crude CFR for all age groups (J) increased at an early stage of the epidemic, and once peaked around April 25 and was followed by an increase in CFR. There is an increasing CFR trend across most age groups, starting around May 10, 2020. Our model-based posterior estimates for the time-delay adjusted CFR are substantially higher than the crude observed CFR at an early stage. Both values are converging as the epidemic progresses.

**Fig. 4:**
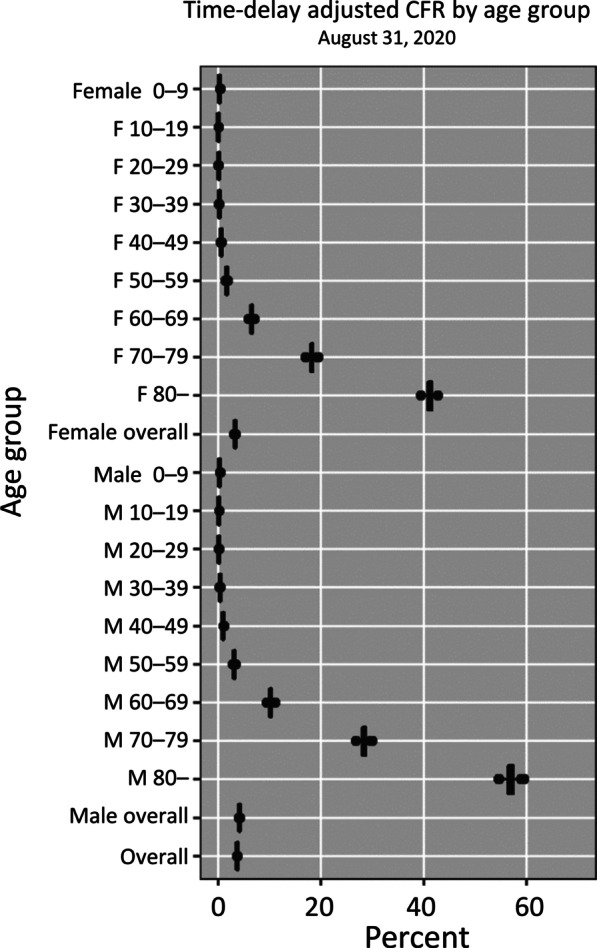
Estimates of time-delay adjusted risk of death caused by COVID-19 by age group and gender, April–August 2020, Chile. Distribution of time-delay adjusted case-fatality risks derived from the latest estimates (August 31, 2020) are presented. Age ranges from top to bottom: 0–9, 10–19, 20–29, 30–39, 40–49, 50–59, 60–69, 70–79, 80 years and older, and all age groups

The time-delay adjusted CFR results by gender and age group, as of August 31, 2020, are shown in Table 2 and Fig. 4. Time-delay adjusted CFR for men and women are 4.16% (95% CrI: 4.09–4.24%) and 3.26% (95% CrI: 3.19–3.34%), respectively, while overall national estimate is 3.72% (95% CrI: 3.67–3.78%). Among men, senior citizens appear to be severely affected; the adjusted CFR is 10.16% (95% CrI: 9.76–10.60%) for men aged 60–69 years, 28.35% (95% CrI: 27.49–29.35%) for those aged 70–79 years, and 56.82% (95% CrI: 55.25–58.34%) for those 80 years old and above. We observe a similar pattern for women. The adjusted CFR is 6.44% (95% CrI: 6.10–6.81%) for women aged 60–69 years, 18.18% (95% CrI: 17.40–18.93%) for those aged 70–79 years, and 41.10% (95% CrI: 40.02–42.26%) for women aged 80 years old or more.

**Table 2:** Summary results of time-delay adjusted case fatality risk of COVID-19 by age group and gender in Chile, 2020 (August 31, 2020)

Age group	Gender	Latest estimate (95% CrI^a^)	Range of median estimates	Crude case fatality rate (95% *CI*^b^)
Overall		3.72% (3.67–3.78%)	3.72–9.94%	3.54% (3.49–3.60%)
Male		4.16% (4.09–4.24%)	4.16–10.20%	3.97% (3.90–4.05%)
Female		3.26% (3.19–3.34%)	3.26–10.19%	3.09% (3.02–3.17%)
0–9	Male	0.23% (0.13–0.34%)	0.17–0.31%	0.21% (0.12–0.32%)
	Female	0.23% (0.14–0.35%)	0.23–0.83%	0.21% (0.13–0.33%)
10–19	Male	0.13% (0.07–0.22%)	0.11–0.53%	0.12% (0.07–0.20%)
	Female	0.06% (0.03–0.12%)	0.04–0.28%	0.06% (0.03–0.11%)
20–29	Male	0.14% (0.11–0.18%)	0.14–0.38%	0.14% (0.11–0.17%)
	Female	0.08% (0.06–0.11%)	0.08–0.92%	0.08% (0.06–0.11%)
30–39	Male	0.33% (0.28–0.39%)	0.33–2.18%	0.31% (0.27–0.37%)
	Female	0.20% (0.16–0.24%)	0.10–0.36%	0.19% (0.15–0.23%)
40–49	Male	0.96% (0.87–1.07%)	0.86–4.42%	0.92% (0.84–1.02%)
	Female	0.59% (0.51–0.67%)	0.32–1.24%	0.56% (0.49–0.64%)
50–59	Male	3.12% (2.94–3.31%)	2.76–7.36%	2.99% (2.81–3.17%)
	Female	1.66% (1.52–1.80%)	1.63–3.02%	1.58% (1.45–1.72%)
60–69	Male	10.16% (9.76–10.60%)	10.16–33.29%	9.69% (9.31–10.10%)
	Female	6.44% (6.10–6.81%)	5.98–27.32%	6.11% (5.78–6.45%)
70–79	Male	28.35% (27.49–29.35%)	27.27–72.74%	26.97% (26.15–27.92%)
	Female	18.18% (17.40–18.93%)	18.16–69.30%	17.25% (16.51–17.96%)
80–	Male	56.82% (55.25–58.34%)	56.82–99.26%	53.62% (52.14–55.05%)
	Female	41.10% (40.02–42.26%)	40.80–97.86%	39.13% (38.10–40.23%)

^a^CrI credibility intervals

^b^*CI *confidence interval

^c^Cumulative cases over cumulative deaths

## Discussion

Few studies have estimated the time-delay adjusted CFR by age group for COVID-19 in Latin America [48], a region with relatively little attention during the ongoing coronavirus pandemic. Consistent with other recent COVID-19 research [31, 33, 49], our results show that the COVID-19 epidemic in Chile has disproportionately affected seniors, especially those aged ≥ 70 years. These results suggest that an aging population could exacerbate the fatality impact of COVID-19 [50], similar to influenza and respiratory syncytial virus [51], and consistent with data available from Italy [50]. The comparatively low CFR observed in Chile during the early stages of the epidemic [29] probably reflected the initial cases' age structure and socioeconomic status. During the first weeks of the pandemic, COVID-19 cases occurred among relatively young age groups, with most transmission occurring among individuals between 20 and 60 years of age, and in high-income communities with better access to healthcare and lower prevalence of risk factors for severe COVID-19 [13, 14, 16, 21, 52, 53].

Our latest adjusted CFR estimates for those aged 80 and over reach values as high as 56.82% (95%CrI: 55.25–58.34) for men and 41.10% (95%CrI: 40.02–42.26%) for women. Adjusted CFR for men aged 80 years of age and older is 2.0-fold higher than our estimates for men aged 70–79 years and 5.6-fold higher than men aged 60–69. Adjusted CFR for women 80 years of age and older is 2.3-fold higher than our estimates for women aged 70–79 years and 6.4-fold higher than women aged 60–69.

The large majority (68.4%) of COVID-19 cases occurred in Greater Santiago during the study period. As seen for the overall population starting in April until about day 75 (June 14), the upward trend in the crude CFR suggests that viral transmission was spreading to more vulnerable populations. Interestingly, throughout March, most transmissions occurred among relatively young, better-off populations. In Greater Santiago, localized lockdowns were put in place starting March 28 through mid-April in seven municipalities, six of which are among the richest in Chile [54]. Viral transmission moved towards lower-income municipalities during April [16, 21]. Among these economically disadvantaged populations, social distancing measures are harder to comply with due to a higher proportion of the population participating in the informal economy, greater population density and overcrowding, more deficient sanitary infrastructure, and lower quality healthcare [14, 21, 55]. Estimates of human mobility during the city-wide lockdown based on cell-phone data across municipalities show that the lockdown was less effective in lower-income municipalities [14, 56]. The higher the rate of multidimensional poverty (based on education, health, labor, housing, and social capital), the less effective was the lockdown in reducing mobility, with up to a three-fold difference in mobility reductions between rich and poor municipalities in Santiago. The disproportionate impact that the COVID-19 pandemic has had on lower-income or more vulnerable populations has also been documented elsewhere [5, 57, 58].

An upward trend in the crude CFR could also have resulted from an increasing number of unreported cases due to a saturated testing capacity [55]. Chile's testing capabilities were greatly expanded during the epidemic, partly as a coordinated effort from the Ministry of Science to include testing from public and private laboratories, including universities that converted research labs to PCR testing labs. Processing capacity grew from a few hundred tests per day to up to 35 000 per day (cumulative of about 130 tests per 1000 population) so far [25]. Despite this massive increase in testing capabilities, the 7-day average test positivity (positive tests/total tests) reached 33% nationally and 48% in Greater Santiago during the mid-June peak of the epidemic. This dramatic increase in positive test rates suggests there may be a substantial proportion of under-diagnosed COVID-19 cases in Chile, at least during the peak of transmission in Greater Santiago [59].

The health system capacity also reached saturation levels during the pandemic's peak by the end of June, with 95% intensive care unit occupancy in Greater Santiago and 89% at the national level [23, 24]. A major disease outbreak, such as COVID-19, can strain the healthcare system and hospital resources [19]. This excess demand may potentially degrade treatment quality or lead to sub-optimal treatment decisions, such as diversion from hospitalization or early discharge, and cause delays in laboratory work and reporting, as has been observed in other public health emergencies [60–62]. However, our data do not allow us to test this hypothesis.

The downward trend in the adjusted CFR at the early stage of the study period may have been influenced by reporting delays. The observed differences in our crude and adjusted CFR estimates are directly due to the time-delay, which we assume fixed during the epidemic.

The small proportion of men (39.2%) among COVID-19 cases in people aged 80 and over is probably attributable to the relatively small male population size for that age group; men represent only 37% of the population > 80 in Chile [63]. Life expectancy at birth in Chile is 77.2 years for men and 82.1 years for women. Life expectancy at age 60 is 21.8 years for men and 24.9 years for women. China and the United States have reported higher mortality among men [64, 65]; our data provide the opportunity to examine the CFR by gender and age.

Our study has limitations. First, our CFR estimates are probably affected by under ascertainment, as has been estimated elsewhere [33, 37, 38]. Under ascertainment may have pushed our estimates upwards if the surveillance system captured the most severe cases. This bias may be particularly problematic during the peak of the epidemic from mid-May through the end of June. Infectious diseases with a substantial share of asymptomatic or mild infections, such as COVID-19, may be more accurately characterized by infection-fatality risk (deaths / infected people). However, those data are not yet available in Chile. Second, we used laboratory-confirmed and probable COVID-19 cases in our analysis, which may result in an overestimation of COVID-19 cases and thus may have pushed our CFR estimates downwards. The demand for diagnostic tests exceeded laboratory capacity in Chile during the peak of the epidemic, as suggested by high rates of positive tests [16]. Test positivity reached a 7-day average of 33% nationally and 48% at the peak of the epidemic in Greater Santiago, suggesting that a substantial proportion of cases were left without testing. In our favor, the circulation of other respiratory viruses has been of no importance during the COVID-19 pandemic in Chile and elsewhere [26] in the southern hemisphere.

## Conclusion

Using real-time epidemiological data from a high COVID-19 testing setting in Latin America, we found that the COVID-19 epidemic in Chile has disproportionately affected seniors, especially those aged ≥ 70 years, suggesting an aging population could exacerbate the death toll brought by the emergence of SARS-CoV-2. COVID-19 is already imposing a high death toll in Latin America. These real-time estimates may help inform public health officials' decisions in the region and underscore the need to implement more effective measures to ameliorate mortality, especially among those facing the highest risk of death.

## Data Availability

The datasets used and analyzed during the current study are available from Base de Datos COVID-19 repository, http://www.minciencia.gob.cl/covid19.
